# Hydrogels for the Delivery of Plant-Derived (Poly)Phenols

**DOI:** 10.3390/molecules25143254

**Published:** 2020-07-16

**Authors:** Nicola Micale, Andrea Citarella, Maria Sofia Molonia, Antonio Speciale, Francesco Cimino, Antonella Saija, Mariateresa Cristani

**Affiliations:** Department of Chemical, Biological, Pharmaceutical and Environmental Sciences, University of Messina, Viale Ferdinando Stagno D’Alcontres 31, I-98166 Messina, Italy; nmicale@unime.it (N.M.); acitarella@unime.it (A.C.); mmolonia@unime.it (M.S.M.); specialea@unime.it (A.S.); fcimino@unime.it (F.C.); mcristani@unime.it (M.C.)

**Keywords:** hydrogels, polymers, physiochemical properties, natural compounds, biological activity, polyphenols

## Abstract

This review deals with hydrogels as soft and biocompatible vehicles for the delivery of plant-derived (poly)phenols, compounds with low general toxicity and an extraordinary and partially unexplored wide range of biological properties, whose use presents some major issues due to their poor bioavailability and water solubility. Hydrogels are composed of polymeric networks which are able to absorb large amounts of water or biological fluids while retaining their three-dimensional structure. Apart from this primary swelling capacity, hydrogels may be easily tailored in their properties according to the chemical structure of the polymeric component in order to obtain smart delivery systems that can be responsive to various internal/external stimuli. The functionalization of the polymeric component of hydrogels may also be widely exploited to facilitate the incorporation of bioactive compounds with different physicochemical properties into the system. Several prototype hydrogel systems have been designed for effective polyphenol delivery and potential employment in the treatment of human diseases. Therefore, the inherent features of hydrogels have been the focus of considerable research efforts over the past few decades. Herein, we review the most recent advances in (poly)phenol-loaded hydrogels by analyzing them primarily from the therapeutic perspective and highlighting the innovative aspects in terms of design and chemistry**.**

## 1. Introduction

**Figure 1:**
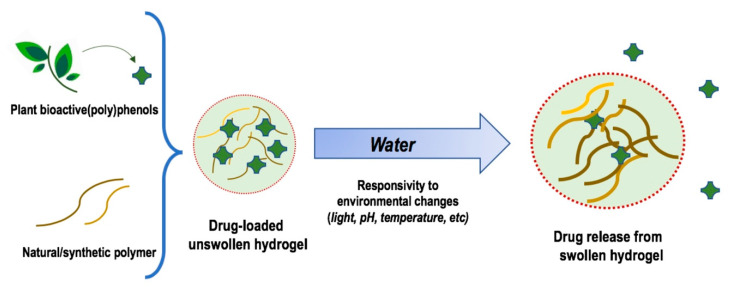
(Poly)phenol-loaded hydrogels as smart drug delivery systems**.**

Hydrogels (HGs) are soft 3D materials capable of swelling in aqueous media and retaining a large amount of water without dissolving. They are also defined as semi-solid systems which resemble in their properties natural living tissue [1]. Basically, a hydrophilic and hyper-branched polymer chain is responsible for the water adsorbing properties, whereas a network of cross-links prevents the solubilization and imparts stability to the resulting system (Figure 1). Being the human body composed mostly of water, HGs are suitable systems for a wide variety of biomedical applications including tissue engineering, biomedical devices, biosensors, drug delivery systems, hemostasis bandages, etc. [2]. Therefore, HGs are commonly and more appropriately defined as “biomaterials”.

There are several ways to classify the HGs. One of the simplest is based on the composition of the polymeric backbone: (i) natural HGs, usually polysaccharides and polypeptides; (ii) synthetic HGs such as polyacrylic acid and polyacrylamide; (iii) co-polymeric HGs; (iv) homo- or multi-polymeric HGs [3]. Natural HGs are generally highly biocompatible and biodegradable but are endowed with poor mechanical properties. On the contrary, synthetic HGs are more stable but might result in cytotoxicity. Co-polymeric backbones are designed to match both biocompatibility and mechanical strength requirements of the desired HGs. Homo-polymeric HGs are referred to as polymeric networks derived from a single monomer, whereas multi-polymeric HGs are an important class of HGs wherein independent cross-linked polymer components form the network (also named double/semi cross-linked interpenetrating network HGs). HGs may form micelles, films and particles. On the basis of the size of the latter, HGs can also be classified as microgels (1 μM to 100 nM), nanogels (< 100 nM) and quasi-nanogels (slightly larger than 100 nM) [4]. The particles size of HGs determines their potential use, especially as drug carriers for biomedical and pharmaceutical applications, with systems in the nanoscale range emerging as the most suitable [5]. Entrapped into the polymeric network, nano-HGs may also contain other nanostructures/nanoparticles. In this case, they are called “nanohybrids” or “nano-functionalised HGs”. Such nanoparticle-HG composites are frequently obtained by the embedment of metal/metal oxide nanoparticles into the HGs matrix to improve the mechanical, biological and physicochemical properties of the pristine HGs, in principle by increasing surface area and stability [6]. The nature of the cross-links along with the 3D network is also of the utmost importance in establishing the potential use of HGs. Chemical linkages, i.e., covalent bonds, are quite stable. Some of them may undergo enzymatic cleavage (ester or amide bonds) within specific biological environments and then HGs containing such linkages may be exploited for target-based or site-directed drug delivery systems [7]. Physical linkages (ionic bonds, hydrogen bonds, van der Waals interactions) are particularly required for the preparation of stimulus-responsive HGs (e.g., thermo- or pH-responsive systems) [8] (Figure 1). HGs may also be defined as neutral, anionic, cationic or amphiphilic according to the overall charge of the 3D polymeric network or its physicochemical properties, respectively.

The term (poly)phenols indicates a group of chemical substances that encompass an aromatic ring bearing one or more hydroxyl substituents or groups, with functional by-products (esters, methyl ethers, glycosides, etc.). The (poly)phenol family represents one of the most abundant and extensively studied class of molecules commonly distributed in the plant kingdom. These compounds are plant secondary metabolites of plants and have a role in the attraction of pollinators, defense against ultraviolet radiation and plant protection against microbes and herbivores. This class of compounds comprises innumerable and highly diversified phenolic structures, including rather small molecules, such as phenolic acids, as well as complex polymerized molecules. (Poly)phenols are considered the main class of phytochemicals with health-promoting properties [9].

The focus of the present review is that of underlining the recent growing interest of the HGs as delivery systems of bioactive plant-derived (poly)phenols, whose use as therapeutic agents and development as drugs is hampered by their limited bioavailability and water solubility. The vast majority of applications in this regard are related to the topical administration of (poly)phenolic compounds due to their extraordinary range of biological properties, including antimicrobial, antiviral, antioxidant, free-radical scavenging, anti-inflammatory, anticancer, etc. However, with the proper combination of polymers, HGs suitable for local injections and oral administration might also be obtained.

## 2. Potential Biomedical Applications of (Poly)Phenols Loaded-Hydrogels

### 2.1. Skin and Epithelial Diseases

A number of papers have reported the design of plant (poly)phenol-loaded hydrogels as potential candidates for the treatment of alterations and pathologies at the level of epithelial tissues, which represent the interface between the body and the surrounding environment.

Skin tissue is a complex structure composed of multiple cell types, and, due to the external exposure, is frequently affected by different stimuli such as thermal and physical insults or chemical agents. The barrier function of the skin is primarily provided by the stratum corneum (SC), the outermost layer of the skin [10]. Generally, excised skin from several animals is utilized to evaluate (trans)dermal drug delivery in vitro. Although excised human skin is clearly the most relevant model, it is often not feasible. Pig skin is recognized as the most appropriate animal model due to the numerous similarities with human skin; however, rat and mouse skin models are frequently used as valuable alternatives in percutaneous absorption studies [11].

Several studies have evidenced that HGs could be efficient delivery systems to improve the skin penetration of plant-derived bioactive compounds such as quercetin (Q) [12], rutin (RU) [13], naringenin (NR) [14], liquiritin (LQ) [15], and ferulic acid (FA) [16]. In particular, stimuli-responsive HGs may be useful in the treatment of skin and epithelial diseases, with pH/temperature-responsive HGs that may be finalized to the physiological microenvironment at the skin site, and light-responsive HGs that can be modulated according to the light’s intensity and wavelength.

#### 2.1.1. Skin Wound Healing

Wounds are acute or chronic interruptions in skin epidermis or mucous membranes [17]. The healing process of acute wounds (such as those created by trauma) in adult mammals, including humans, progresses in four orderly phases that overlap in time: coagulation, inflammation, proliferation, and remodeling. However, much of the molecular mechanisms involved in this process are poorly understood. The healing process is influenced by several factors, especially size, depth, and degree of injury. The deregulation or interruption of one or more phases of the normal healing process lead to chronic wounds.

One of the major causes of impaired wound healing includes diabetes mellitus. In fact, impaired wound healing is one of the prevalent long-term complications of diabetes mellitus [18]. Diabetics often develop open, non-healing ulceration of the toes, foot or leg and are more prone to wound complications, since in these patients the wound healing mechanism is adversely affected due to the impairment of angiogenesis and neovascularization.

Several animal models of acute and chronic healing have been developed, especially in rodents, and can provide valuable information about efficacy of potential therapeutic products [19]. Each of these models has undoubted advantages (cost, availability, similarity to humans, etc.). but also evident limitations, due to significant anatomical and physiological differences among species. For example, rat and mouse skin is characterized by the existence of a subcutaneous striated muscle layer called the panniculus carnosus, producing rapid wound contraction following injury, while in humans wound healing is dominated by reepithelialization and granulation tissue formation. Due these differences, the results obtained in preclinical studies using animal models of wound healing must be interpreted with great caution before transposing them to a clinical context.

Wound dressings could accelerate the healing process [20]. Recent dressings are characterized by improved biocompatibility and humidity retention, thus improving the hypoxic environment. In particular, HGs are used to treat different kinds of wounds with minimal-to-moderate exudate (e.g., surgical wounds, burns, radiation dermatitis, etc.). In fact, owing to their excellent moisturizing ability, HGs maintain the proper moisture rate at the wound site and play a positive role in the cleansing of necrotic tissue. Some ingredients used to prepare HGs may themselves have properties promoting wound healing. For example, chitosan (CS), besides biocompatibility, biodegradability and tissue-adhesive properties, showed hemostatic and anti-infective activity, and can stimulate macrophages to produce growth factors (GFs) with positive effects on extracellular matrix production [21]; gelatin (G) has been used clinically as a wound dressing because of its richness in amino acids [20]; hyaluronic acid (HA) hydrates and modulates the cellular microenvironment, and contributes to the orientation of the extracellular matrix and fibrous component; its cell surface receptor bindings induce cell-to-cell adhesions, cell–substrate adhesions, proliferations, and cell migrations [21]. In addition, the degradation rate of the HGs can also be adjusted, and thus this biomaterial is appropriate for use as a carrier of bioactive agents with healing, anti-inflammatory, and/or antioxidant potential efficient for treatment of wounds [20]. In this regard, several naturally occurring (poly)phenols such as the flavonoids RU and Q, the phenolic acids chlorogenic acid (CGA) and FA, the carotenoid curcumin (CU), etc., are known to possess an extensive range of therapeutic activities also useful for wound healing, due to their free radical scavenging, antioxidant, lipid peroxidation inhibitor, immunomodulatory, angiogenic, antibacterial and neurogenic properties [22,23]. In spite of possessing important pharmacodynamics features, these compounds have encountered potential limitations in the therapeutic use due to their hydrophobic nature and poor solubility in aqueous solution. For example, Q is a promising topical healing agent because it can increase fibroblast proliferation, decrease immune cell infiltration, and modulate fibrosis-associated signaling pathways. However, it has a very limited ability to penetrate the skin; although it is insoluble in water and has a favorable lipophilic partition coefficient due to the nonpolar groups in its structure, the polar hydroxyl groups hinder skin penetration. Thus, HGs ensuring a high loading capacity and adherence to the skin represent an interesting option to overcome these issues and obtain sustained drug levels at the tissue target.

HGs to vehicle RU [24,25], CGA [26], Q [27], FA [28], and oligomeric proanthocyanidins (OPC) [29], as well as polyphenol-rich plant extracts [30,31], appeared effective in ameliorating wound healing in in vivo rat and mouse models, and also in animals with diabetes mellitus experimentally induced by streptozotocin or alloxan (Table 1) [28,29,30,31]. An innovative RU-conjugated CS-HG was projected by Tran N.Q. et al. as a type of injectable wound dressing for dermal wounds [25]; this RU-conjugated CS-PEG-tyramine (RU-CS-PEG-TY) polymer in the presence of horseradish peroxidase (HRP) and H_2_O_2_ forms an in situ gel, able to enhance wound healing on male Sprague Dawley rats. Recent papers demonstrated the possibility to use HGs while taking advantage of the synergic interaction occurring among the intrinsic healing properties of different components. For example, Jee J.-P. et al. studied in a diabetic mouse model a Carbopol HG system for topical use containing skin-permeable GFs, Q and oxygen carriers in order to increase re-epithelialization and granulation tissue of diabetic wounds, by rising the concentrations of GFs and antioxidants, and the oxygen delivery in the wound acting in a synergic way [32]. Gallelli G. et al. evaluated both the clinical efficacy and the safety of a HA nano-HG embedded with Q and oleic acid (an unsaturated fatty acid known to accelerate the wound healing process) in the treatment of lower limb skin wounds in fifty-six patients with diabetes mellitus and unsuccessfully treated with mechanical compression [33].

A peculiar problem is represented by burn injuries that necessitate the development of specific therapeutic materials endowed also with antibacterial activity against infectious microbes prevalent in burns [59]. A thymol enriched bacterial cellulose HG (BCT-HG) appeared suitable as burn wound dressing material, being effective against *Escherichia coli*, *Staphylococcus aureus*, *Pseudomonas aeruginosa* and *Klebsiella pneumoniae* and possessing in vivo good wound healing property in rats bearing third degree burn wounds [34]. However, some natural polyphenols are used as components of HGs for purposes different from the bioproperties mentioned before. For example, OPC were examined as a natural cross-linking reagent to make poly-γ-glutamic acid/gelatin (γ-PGA)/G) HGs, able to accelerate wound contraction and re-epithelialization in an in vivo wound healing model in rats [60].

#### 2.1.2. Epithelial Pathologies

One of the most common categories of inflammatory skin disorders is contact dermatitis, which may be classified as allergic and irritant contact dermatitis (ICD), being characterized as eczematous lesions caused by exogenous sensitizing or irritating agents [61].

According to Rigon C. et al. [35], a Pemulen^®^ TR2 HG containing silibinin (SB)-loaded pomegranate oil-based nanocapsules could be useful for cutaneous application in ICD, as demonstrated in croton oil-induced ear edema in male Swiss mice. Shrotriya S.N. et al. [36] designed SB-loaded solid lipid nanoparticle-enriched Carbopol gel whose topical application appears efficient in dinitrochlorobenzene-induced ICD in mice. Szekalska et al. [62] tested an alginate-based HG, considered a suitable drug carrier for this kind of application due to its antiallergic and anti-inflammatory activities, incorporated with cynaroside (CYN) [a luteolin (LT) derivative]. Theses HGs were efficient for the treatment of atopic dermatitis, as demonstrated not only in a carrageenan-induced mouse paw edema inflammation model but also in an oxazolone-induced mouse allergic contact dermatitis model. One has to remember that polyphenols can form insoluble complexes with allergenic proteins, changing their structure or rendering them less bioavailable, and can affect different phases of the allergy process.

Excessive exposition to sunlight UV radiation (both UVA, 320–400 nm, and UVB, 280–320 nm) can lead to skin structure changes, cell injury, premature skin aging with direct DNA or skin damage. UVB radiation induces a number of pathologic changes, including skin inflammation with vasodilatation, edema, erythema and epidermal hyperplasia. Marchiori M.C.L. et al. showed that a gellan gum-based HG containing SB and pomegranate oil is able to protect against UVB-induced ear edema in male Swiss mice [63], and Balestrin L.A. et al. demonstrated that a Carbopol^®^ Ultrez 20-based HG containing an extract from *Achyrocline satureioides* rich in Q and LT is able to protect porcine ear skin against in vitro UVA/UVB-induced damage [39].

Oxidative damage to the cornea, such as that induced by alkaline chemical burn, may cause vision loss or blindness. However, ocular topical application of antioxidant compounds showed very low bioavailability and short contact time, thus representing an important limitation of this approach. The results of the study by Tsai C.-Y. et al. suggest that a thermo-sensitive CS-HG containing FA, demonstrated to be effective in a rabbit corneal alkali burn model, may provide an alternative to traditional eye drops in the treatment of this pathological condition [40].

Psoriasis is characterized by erythematous skin lesions. Although the cause of psoriasis has not been elucidated yet, hyperproliferation and abnormal differentiation of epidermal keratinocyte and elevated inflammatory responses have been reported. In normal skin, the pH should be maintained at about 5.5, but in psoriatic skin, this pH value may rise to 6.0 or higher. Recently, HGs with stimulus-responsive ability have attracted much attention (Table 1). HA has carboxyl groups and therefore it can ionize at pH 6–7 and release the carried drug by electrostatic repulsion, so that HA is often used in pH-sensitive drug delivery systems. A HG composed of temperature-sensitive poly(*N*-isopropylacrylamide) and HA loaded with LT could be an interesting candidate for psoriasis treatment, since it can release the drug easier in the basic environment of psoriatic skin [37].

**Figure 2:**
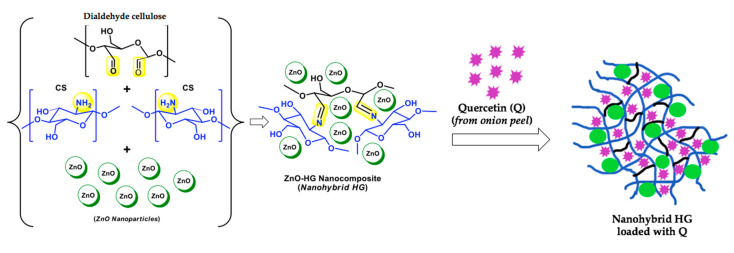
Schematic representation of the nanocomposite hydrogel (HG) developed by George D, et al. [42]; base polymer formed by chitosan (CS; drawn in blue) cross-linked with dialdehyde cellulose (drawn in black) via Schiff base formation (highlighted in yellow). ZnO nanoparticles marked in green and quercetin (Q; from onion peel waste) marked in purple.

In particular conditions, such as minor trauma, impaired host immunity, or environmental factors, an increased risk of developing skin and epithelial infections has been observed. Several plant compound-loaded HGs appeared suitable for dermatological applications, since they are safe for skin cells but toxic for bacterial and fungal strains (Table 1). For example, cyclodextrin(CD)-based HGs loaded with caffeic acid (CA) have been reported by Pinho E. et al. as effective against *Staphylococcus epidermidis*, *Staphylococcus aureus* and *Klebsiella pneumoniae* [41]. Interestingly, George D. et al. developed a nano-functionalized hydrogel prepared by embedment of ZnO nanoparticles (CHGZ-HGs) so that enhanced surface area and stability rendered by the nanoparticles in the polymer matrices improved the carrier functionality towards efficient drug loading and delivery (Figure 2). In particular, they demonstrated that onion peel Q-CHGZ-HGs, with increased drug loading of 39% vs pure chitosan HGs, are effective against *Staphylococcus aureus* and *Trichophyton rubrum* [42].

Periodontal tissue damage and inflammation are initiated by bacteria throughout every stage of the disease, so it may be ameliorated by the local delivery of antimicrobial, antioxidant and anti-inflammatory drug. However, the mucous membranes do not possess a keratinized outer layer as for the skin and are therefore much more sensitive to irritants. Thymol-loaded CS-HGs are reported to be effective against *Streptococcus mutans* and *Staphylococcus aureus*, and so possess desirable properties for a mucosal delivery system for therapy of periodontal disease [43].

Recurrent aphthous stomatitis (RAS) is a common condition in which ulcers recur on the oral mucosa. It is one of the most painful oral inflammatory ulcerative conditions, causing pain during eating, swallowing and speaking. Topical treatment is used to promote healing and pain relief, and topical medications with mucosal adherence properties have been used with some success. The effectiveness of patches made from tragacanth gum to control the pain and reduce the healing time of RAS in humans was increased by addition of licorice (*Glycyrrhiza glabra*) extracts due to the well-known anti-inflammatory properties of this ingredient [44].

#### 2.1.3. Epithelial Cancer

Melanomas are malignant tumors derived from melanocytes, and represent one of the leading causes of premature death from cancer. The most common site of involvement is the skin. Melanoma is resistant to radio-/chemotherapy and the main treatment is surgical resection. However, the potential residual melanoma cells may cause cancer recurrence, and the large wounds resulting from melanoma resection are difficult to heal and constitute a main issue for the therapeutic treatment.

A Q-CHGZ-HG showed the maximum drug release at pH lower than 5.0 and, working in an acidic environment, would be suitable for anticancer applications, as demonstrated on human skin carcinoma A431 cells [42]. A thermo-responsive HG based on functionalized caprolactone-PEG and loaded with SB, having a sol-gel transition temperature of 34–35 °C, appeared endowed with a great potential for local treatment of malignant melanoma, as shown on B16-F10 melanoma cells both alone and in combination with doxorubicin [38]. Taking into account the photothermal property of OPC, OPC-containing HG scaffolds, based on calcium silicate nanowires and sodium alginate, were demonstrated by Ma H. et al. to function as a natural photo-thermal agent for melanoma therapy in a Balb/c mice bearing B16-F10 tumor model [29]. In fact, using near infrared (NIR) laser irradiation, the high temperature induced by OPC-containing HG scaffolds could kill melanoma cells and suppress tumor growth. Huang Z. et al. developed HGs using PEG-based polymers modified with boronic acids as backbones and ellagic acid (EA); the HG is formed at physiological conditions via in situ gelation at pH 7.4. EA release from HG significantly affected CAL-27 human oral cancer cells viability [45].

### 2.2. Injectable and Targeted Hydrogels

Injectable HGs have received more attention relating to cell therapy and tissue regeneration resulting from the applications in minimally invasive surgical procedures (Table 1). These HGs can be injected in situ, forming a desired shape and adhering to the nearby tissues during HG formation.

Traumatic brain injury (TBI) is an extremely serious neurological disorder and the inhibition of oxidative stress following TBI could effectively protect the brain from further impairments. An injectable thermo-sensitive chitosan/gelatin/β-glycerol phosphate (CS/G/GP) HG for the controlled release of the phenolic antioxidant FA was projected by Dong G.-C. et al. and tested on Neuro-2a cells exposed to H_2_O_2_; thanks to the gelation temperature of 32.6 °C, C/G/GP HG was demonstrated to be a clinically efficient system [46].

The nucleus pulposus (NP) is the central part of the intervertebral disc, and it is mainly constituted of water and type II collagen, together with proteoglycans and small cartilage-like cells interspersed. Disc degeneration is generally believed to originate in the NP and can be accelerated by oxidative stress induced by reactive oxygen species (ROS) able to induce the apoptosis of NP cells. A thermo-sensitive chitosan/gelatin/glycerol phosphate loaded with FA (FA-CS/G/GP) HG was formulated for gradual release of FA to treat NP cells from the oxidative stress-induced damage, since the gelation temperature of the FA-CS/G/GP HG was 32.17 °C. The results obtained on NP cells exposed to H_2_O_2_-induced oxidative stress suggest that this thermo-sensitive HG may have potential application for NP regeneration [47].

Oxidative stress plays a main role also in osteoarthritis (OA), a degenerative joint disease characterized by cartilage loss, synovial inflammation and subchondral bone remodeling, which leads to pain and joint stiffness. Intra-articular therapy provides a direct route of drug administration to a diseased joint and has many advantages, including increased bioavailability and minimized potential systemic side effects. A thermo-sensitive, biodegradable in situ-forming HG, containing Q and formed by a methoxy-PEG-l-poly(alanine) (mPEG-PA) polymer, was shown to be able to reduce cartilage degradation in rats which had undergone anterior cruciate ligament transection [48].

Aging-related oxidative stress is considered a major risk factor of cardiovascular diseases (CVD). Taking into account that Cisd2 is an outer mitochondrial membrane protein whose deficiency can cause mitochondrial breakdown and cell death, a thermo-sensitive CS-gelatin-based HG containing FA (FA-CS/G-HG) was projected by Cheng Y.-H. et al. and tested in vitro on Cisd2-deficient (Cisd2^−/−^) cardiomyocytes (CMs) under oxidative stress and in vivo in intramyocardially injected Cisd2^−/−^ mice. Furthermore, this HG can form a gel at body temperature, suggesting potential applications of this thermo-sensitive FA-loaded HG in the treatment of CVD [49].

CD44 is a cell membrane-bound surface receptor that mediates cell–cell and cell–extracellular matrix (ECM) communication. It is associated with tumor progression, and has been found to be overexpressed in numerous cancer types, including breast, lung, and glioblastoma. Since HA, a major constituent of the ECM, is one ligand of CD44, it has been used in active targeting on CD44-overexpressing cancers, increasing drug uptake, and therapeutic efficacy. Barbarisi M. et al. tested a nano-HG of HA loaded with the flavonoid Q [50], in order to improve its GBM cell internalization and increase the cytotoxicity of the alkylating drug temozolomid, as studied in two GBM cell lines with different O638-methylguananine-DNA methyltransferase (MGMT) expression levels (A172 and T98MG cells, having low and high MGMT expression levels, respectively). The aim of the study from Quagliariello V. et al. was based on the CD44 targeting by using HA nano-HG of Q tested alone and in combination with Everolimus in hormone-responsive human breast cancer MCF7 cells [51]. Similarly, the authors tested the use of a HA-HG of Q alone and in combination with SNS-314 (an inhibitor of Aurora Kinase type A and B) on human medullary and papillary cancer thyroid cells (B-CPAP and TT cell lines) [52].

### 2.3. Hydrogels for Oral and Systemic Administration

As mentioned before, stimulus-responsive HGs are three-dimensional polymers able to experience rapid modifications in their microstructure, following small changes in the environment. Furthermore, the responsivity to environmental changes (e.g., temperature, pH, electric and magnetic fields) makes them extremely attractive as smart drug delivery systems, due to their ability to release drug molecules on demand. Among these systems, pH-responsive HGs have been broadly explored in different biomedical areas and tested for targeted drug delivery and as carriers of easily degradable bioactive molecules (Table 1).

In fact, pH-responsive properties of HGs are mandatory for their potential use as oral drug delivery systems, also targeting specific areas of the gastrointestinal (GI) tract. Drugs used for oral administration may be totally or in part destroyed in the gastric milieu or absorbed from the upper GI tract before reaching the proximal colon, thus needing the use of higher doses, which may result in several unfavorable effects. For example, Zhao X. and Wang Z. prepared a gellan gum HG as a carrier of apigenin (APG). In vitro release studies demonstrated that the composite HG could control the drug release under acidic (pH 1.2) and weak alkaline conditions (pH 7.4), due to the strengthening of HG networks in an acidic medium and their erosion in a weak alkaline medium [53]. Hu X. et al. developed a novel polyelectrolyte complex (PEC) HG, prepared by self-assembly of two food-grade polysaccharides salecan and *N*,*N*,*N*-trimethyl CS. Green tea polyphenols (GTP) were efficiently encapsulated into PEC HGs and liberated in a sustained pattern, since the amount of GTP released in simulated intestinal fluid (SIF, pH 1.2) was significantly higher than that released in simulated gastric fluid (SGF, pH 6.8) [54]. However, some natural polyphenols have been used as components of HGs and not for their healthy properties. For example, a pH-responsive HG synthesized by the coupling reaction of polyacrylic acid and catechin was proposed as a carrier of oxidable drugs toward the GI tract [64].

The development of pH-sensitive HG is also considered a good approach for colon-targeted delivery of drugs, as in the case of ulcerative colitis, a chronic inflammatory disorder of the GI tract. A RU-loaded poly(starch/acrylic acid) HG showed a strong pH-dependent release behavior, thus offering a maximum release as pH increased from pH 6.8 (small intestine pH) up to pH 7.7 (colon pH), and is endowed with a good protective effect after oral administration on mucosal injury in a dextran sulphate sodium-induced rat model of colitis [55].

Many serious chronic diseases of the small intestine, such as ulcers and even carcinogenesis, have been demonstrated to be related to epidermal infections. It is difficult to maintain sufficient drug concentrations for long periods in the small intestine. Epigallocatechin gallate (EGCG) and amyloid fibrils-based HGs showed strong toxicity against Gram-(+) bacteria (*Listeria monocytogenes*, methicillin resistant *Staphylococcus aureus* and *Streptococcus oralis*) and Gram-(−) bacteria (*Escherichia coli*, *Klebsiella pneumoniae*, and *Pseudomonas aeruginosa*), without any notable cytotoxicity to human colonic epithelial cells. This system seems to possess a good application potential in diseases related to the infection of the small intestine, since at that pH (around 6), it could coat the epidermal surface and exert its antibacterial activity [56].

The pH-sensitive HGs also could have importance for the oral administration of cancer therapeutics, with safe and controlled delivery for various solid tumors. Recently, Kunjiappan S. et al. studied an orally delivered pH-responsive Zein-*co*-acrylic acid HG incorporated with dual-drug (5-fluorouracil (5-FU) and RU) for improved anticancer properties with less toxicity. This system, able to arrest the proliferation of MDA-MB-231 and MCF-7 breast cancer cell lines, was optimized to improve its drug loading and encapsulation efficiency, and the release at pH 1.2 and pH 7.4 [57].

Inhibition of amyloid β-protein (Aβ) aggregation is considered as a promising strategy for the prevention and treatment of Alzheimer’s disease. Since conjugating CU to HA can increase CU solubility, Jiang Z. et al. demonstrated that EGCG-CU bi-modified HA (CEHA) can self-assemble into nanogels, thus developing a potent nano-inhibitor on Aβ aggregation and cytotoxicity; interestingly, EGCG and CU are both effective inhibitors of Aβ aggregation, but acting through different mechanisms, as shown on SH-SY5Y neuroblastoma cells incubated with Aβ [58].

## 3. Hydrogels Projected for Topical Applications

### 3.1. Quercetin-Containing Hydrogels

**Figure 3:**
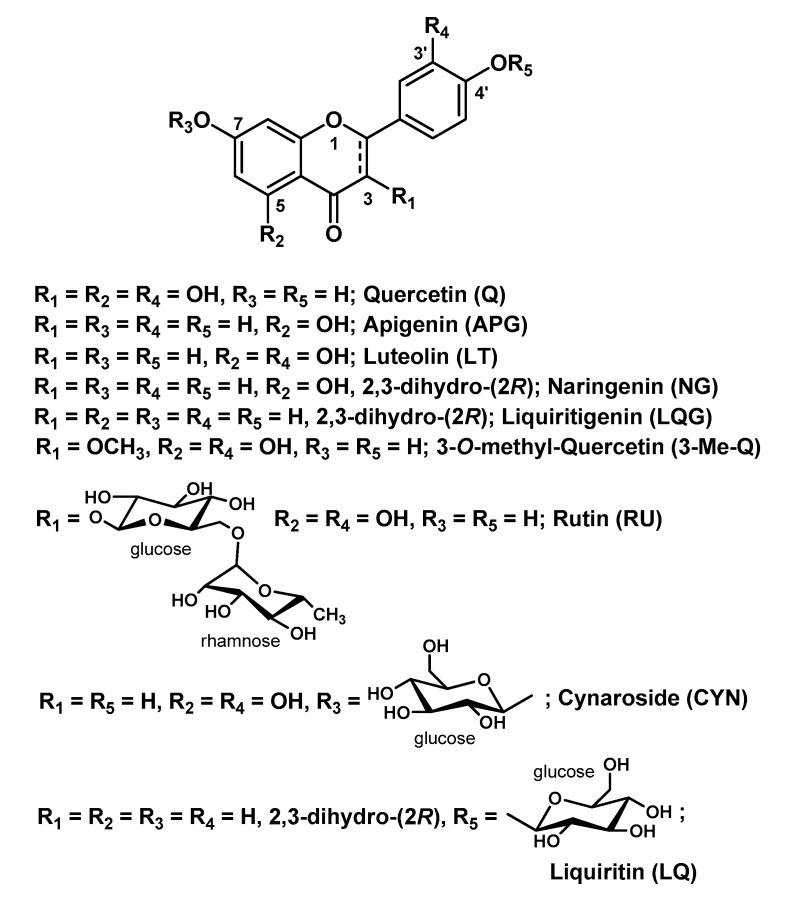
Chemical structure of quercetin (Q) and some related natural occurring flavonoids.

Q (3,5,7,3′,4′-penthahydroxyflavone; Figure 3) is a flavonoid (sub-class of flavonols) found in many fruits and vegetables. Chemically, it is a polyphenolic compound bearing a C6-C3-C6 backbone. Q, as well as many other flavonoids, has a wide range of healthy and pharmacological effects. Unfortunately, the employment of flavonoids is restricted by their limited solubility in water, chemical instability, low permeability through biological barriers and poor oral bioavailability.

The properties of Q, together with the capability of the HGs to load/deliver hydrophobic compounds and provide suitable milieu for natural healing, make Q-containing HGs (Q-HGs) ideal wound dressings for the healing of wounds, especially those associated with chronic disorders. Several research efforts in this regard have been made in recent years to provide the most effective Q-HG system.

Jangde R. et al. proposed a layered HG film composed of Carbopol HG loaded with Q-containing liposomes and G. This multiphase HG system with the optimal ratio G/Carbopol of 6:4 accelerated the wound healing in animal models, both in vitro and in vivo [27]. The advantage of this technology was that of exploiting a polymeric matrix (i.e., Carbopol) with porous morphology, whose hydrophilicity has been enhanced by the incorporation of G, maintaining the required level of moisture (suitable for the management of wound repair) without phase separation. In addition, the addition of G (10%) led to a lamellar structure of the system which shows biochemical similarity with the intact skin and increased delivery potential.

Gallelli G. et al. reported, in a pilot study on patients with diabetic foot ulcer, that a nano-HG of HA embedded with Q and oleic acid significantly reduced the healing time of the skin wounds in comparison to HA, without adverse reactions [33]. The key feature of this system is employing two bioactive components that act synergistically on the target disease and a delivery platform (i.e., HA) particularly suitable to affect the signs of the disease for the above-referred properties.

Another example of HG designed with the aim of exploiting the synergic effect of more components has been provided by Jee J.-P. and co-workers. In this case, they combined a Q-loaded oil-in-water nanoemulsion, a hyperoxygenated nanoemulsion containing 1-bromoperfluorooctane encapsulated in soy phosphatidylcholine, and a combination of four highly skin-permeable GFs genetically fused to low-molecular-weight protamine via the *N*-termini. All components were then incorporated into a Carbopol 981 HG matrix. This system turned out to be effective in the acceleration of the re-epithelialization and granulation tissue formation in diabetic wounds due to the prolonged skin infiltration of the bioactive components [32]. GFs were employed since impaired chronic wound healing is characterized by their deficiency [65]; 1-bromoperfluorooctane has been used as a source of oxygen because tissue repair requires increased metabolic activity, then a high demand of oxygen, which also prevents microbial infections at the wound site, stimulates phagocytes, promotes neovascularization, degradation of necrotic tissue and collagen production [66].

Synergic effects on the antimicrobial activity have been obtained by George D. and co-workers in an ingenious and eco-friendly design of a nanohybrid HG containing Q and ZnO nanoparticles, suitable also for the treatment of skin cancer diseases [42]. ZnO was chosen both for its antimicrobial and UV screening properties. This system was prepared using a Schiff base cross-linking strategy by reacting the free amine groups of CS and the aldehyde functions of dialdehyde cellulose, obtained in turn from sugarcane bagasse cellulose and sodium metaperiodate. This strategy avoided the use of chemical cross-linking reagents which are usually associated with toxicity concerns. CS is a liner polysaccharide derived from crustacean shells by partial alkaline deacetylation of the chitin and is constituted by units of glucosamine and *N*-acetyl-glucosamine. As a polycationic polymer, it possesses its own antimicrobial activity and bioadhesive properties which make it particularly appropriate for topical formulations [67]. The polysaccharidic HG matrix was embedded during its formation with the ZnO nanoparticles, phyto-synthesized by using a green extract of muskmelon seeds as reducing agent and zinc acetate. Q was employed as “phyto-derived Q”, obtained from onion peel waste by microwave-assisted extraction and eventually loaded as the drug into the nanohybrid HG by the swelling method (soaking dried HG in drug solution). This intriguing ZnO-HG nanocomposite showed promising results in terms of biocompatibility, antibacterial, antifungal and anticancer (against skin carcinoma) activity for topical applications. The anticancer activity of this nanohybrid HG is exclusively due to the presence of Q, whose huge potential as an anti-carcinogenic and anti-metastatic drug has been recently reviewed [68].

Schwingel L.C. et al. carried out a comparative study on Q and its 3-*O*-methyl-derivative (3-Me-Q; Figure 3) loaded in two HGs, i.e., CS and hydroxypropyl methylcellulose (HPMC), related to their in vitro skin permeation/retention profile [12]. For both compounds, CS-HG provided the best permeation/retention performance into the three skin layers (stratum corneum, epidermis, dermis) but with Q to a much higher extent compared to 3-Me-Q. Interesting outcomes were derived by adding 5% β-cyclodextrin (β-CD) to both systems as permeation promoter: for Q, the best permeation/retention profile was obtained when it was incorporated into CS-HG containing 5% β-CD, whereas, for 3-Me-Q, the HPMC-HG containing 5% β-CD turned out to be the best formulation. This evident behavior discrepancy cannot be easily justified by an additional methyl group in the chemical structure of Q. It is realistically related, instead, to the different complexation of the bioactive compound with the β-CD, a cyclic oligosaccharide with a truncated cone shape with high capacity to entrap a wide range of molecules within its cavity, used in pharmaceutical technology to sustain/control the release of drug [69]. Therefore, the same authors performed studies of molecular docking and molecular dynamic simulation to rationalize the experimental results. These theoretical studies indicated that the presence of the 3-*O*-methyl group afford more stable inclusion complexes for 3-Me-Q over Q with the hydrophobic cavity of β-CD. Therefore, the release of 3-Me-Q turns out to be delayed with respect to the release of Q.

Superior skin permeability properties for Q have been also demonstrated in comparison with its naturally occurring glycoside RU (also named quercetin-3-*O*-rutinoside; Figure 3), in a two-step delivery system developed by Park S.N. and co-workers [13]. This consists of ceramide liposomes (as biocompatible carriers for both flavonoids) incorporated into cellulose HG obtained in turn by using (±)-epichlorohydrin (ECH) as cross-linking agent. Although RU showed greater encapsulation efficiency into the HG matrix and better in vitro release than Q, the latter provided a faster diffusion rate, which reflected skin permeability. In this study, the HG matrix played a simultaneous dual-role of disrupting the skin barrier by the hydration mechanism (breaching function) and protecting the skin from water evaporation and external insults (shielding function). On the other hand, ceramide liposomes were prepared by using different ratios of cholesterol and oleic acid to obtain a structural motif that resembles the composition of the intercellular lipid. The latter disappears when the skin barrier is disrupted by the HG, enhancing the transdermal delivery of the bioactive molecules and their sequestration within the stratum corneum.

The pore size distribution of a HG matrix is another fundamental parameter for a delivery process. In relation to Q, this issue has been pinpointed by Varghese J.S. et al. in a composite HG G-carrageenan [70]. G is a natural polypeptide widely used in biomedical and pharmaceutical applications because of its low cost, biocompatibility, biodegradability and non-immunogenicity. Moreover, it is particularly useful as a wound dressing, wound healing and hemostatic agent [71]. When added to carrageenan (a family of sulfated polysaccharide extracted from red edible seaweeds), the resulting composite HG showed an increased pore size distribution in comparison with the individual polymers HGs, leading to an improved Q release in vitro. Additionally, this composite system has been demonstrated to have an improved thermal stability with the optimal ratio G-carrageenan 1:0.5.

The use of RU in the place of Q for the development of delivery systems is less common, as all biological properties of the flavonoids are ascribed to the aglycon component and RU does not present better properties in terms of water solubility and bioavailability with respect to Q. Almeida J.S. and co-workers developed a RU-loaded Carbopol^®^ Ultrez 10 NF HG to assess, in an in vivo animal model, the skin wound healing properties of RU [24]. In this HG system, triethanolamine was employed both as neutralizing agent for the gelation process and as networking linker. The obtained HG showed pH and rheological behavior (i.e., non-Newtonian pseudo-plastic flow, with viscosity that decreases by increasing the shear rate) suitable for dermatological formulations. The antioxidant and free-scavenging activity of RU was also assessed, confirming a close correlation between RU concentration in the skin and wound healing process.

Q has also been recently investigated for ophthalmic applications in a smart stimulus-responsive HG by Yu Y. and co-authors [72]. In this research work, Q was loaded into the HG matrix by means of nanostructured lipid carriers, composed of a mixture of Compritol^®^ 888 ATO (solid lipid) and MCT (medium chain triglyceride; liquid lipid), which were designed to facilitate the transcorneal penetration of hydrophobic drugs with respect to standard solid lipid nanocarriers. The Q-loaded nanostructured carriers were then incorporated into a dual-responsive HG composed of carboxymethyl CS (a semi-synthetic, water-soluble and pH-sensitive CS derivative) and Poloxamer 407 [a synthetic and temperature-sensitive co-polymer containing polyethylene oxide (70%) and polypropylene oxide (30%)]. The polymers were joined with the naturally occurring and non-toxic cross-linking agent genipin to afford the dual-responsive HG with the proper mechanical strength. Release studies showed promising results in terms of dual pH (7.4) and temperature (35 °C) responsiveness and sustained release of Q.

The reactive functional groups of Q, i.e., the phenolic hydroxyl groups, have also been exploited for the preparation of “antioxidant polymers”, which are polymers containing antioxidants that undergo in situ controlled release as a consequence of the biodegradation of the polymeric matrix. The main aim of designing this type of polymer is reducing possible inflammatory responses caused by the local accumulation of polymer degradation products. However, these systems could, at the same time, be exploited for the safe delivery of drugs. In this context, Wattamwar et al. developed poly(antioxidant β-amino ester) biodegradable HGs of Q and CU, another naturally occurring polyphenolic plant product with an extraordinary range of biological properties, including antioxidant activity [73]. Both bioactive components were polyacrylated at the -OH groups to afford individual polymeric networks endowed with easily cleavable ester moieties and free α,β-unsaturated carbonyl groups. The two polyacrylates were then blended with a primary diamine (4,7,10-trioxa-1,13-tridecadiamine), which acted as a bifunctional cross-linking reagent by Michael addition reaction to provide the HGs. Cytotoxicity and antioxidant assays, performed on endothelial cells (HUVECs), confirmed that that this functionalized HG system could be used for the local delivery of drugs and tissue engineering applications.

The same two polyacrylates were use as hydrophobic cross-linkers by Tang S. et al. to modulate the physicochemical properties of the homopolymer *N*-isopropylacrylamide, a known component of negative temperature-responsive HGs, which exhibits a reversible phase change from hydrophilic to hydrophobic state at his its low critical solution temperature (LCST; ~33 °C) [74]. Q- and CU-polyacrylate were grafted into the homopolymer through the free radical polymerization method by using ammonium persulfate as an initiator to obtain temperature-responsive co-polymerized HGs with swelling ratio that decreases with increasing the cross-linker content. The exclusive advantage of using these two functionalized polyacrylates is having enough C=C moieties to react with the vinyl groups of the base homopolymer (i.e., *N*-isopropylacrylamide) to avoid the employment of further chemical linkers that might lead to toxic by-products and/or laborious purification processes. Moreover, in both types of co-polymerized HGs, the bioactive polyphenolic component is joined with the acrylic acid units by means of ester moieties that are smoothly cleaved in the biological environment.

### 3.2. Hydrogels Containing Other Flavonoids

Although Q is known as the flavonoid with the best and widest bioactivity profile, other natural flavonoids with the basic skeleton C6-C3-C6 have been employed for the development of HG-based systems for the delivery of such a class of compounds. The range of biological activities of these flavonoids mostly overlaps with that of Q, and some of them proved to be equally effective in topical formulations, especially as antioxidants for skin protection. In this regard, outstanding research efforts have been made by Park’s group which worked on, besides Q [13], another three flavonoids highly related to Q, namely liquiritigenin (LQG), NR and LT (Figure 3). The authors developed a ceramide liposome-cellulose HG system wherein they loaded LQG and its natural glycoside LQ, known as licorice flavonoids [15]. As for their previous Q-based complex system, they loaded the bioactive components into liposomes, which were in turn incorporated into cellulose HGs by the swelling method. A 6% urea solution was employed to solubilize the liposomes in order to increase the water uptake degree of the HGs according to their previous studies [75]. These complex systems turned out to be effective in enhancing the transdermal penetration of both LQG and LQ in comparison with the single systems, and with the glycoside derivative showing better outcomes. They also prepared a pH-responsive HG for the transdermal delivery of NR, one of the major flavonoids of the citrus fruits [14]. This smart-HG system is composed of carboxymethyl cellulose grafted with 2-hydroxyethyl acrylate (cl-CMC-g-pHEA) and cross-linked by PEG diacrylate via radical polymerization using potassium persulfate as radical initiator. The grafting agent 2-hydroxyethyl acrylate, which also polymerizes during the synthetic process of the HG, was successfully employed to improve the mechanical and physicochemical properties (e.g., adhesion, flexibility) of the base polymer. In vitro skin permeation studies validated this smart pH-responsive HG as a potential delivery system for topical applications, in particular for atopic dermatitis, as the system showed greater swelling ratio at pH 7.5 (the acne skin pH) and 8.5 (the atopic skin pH) than at 5.5 (the normal skin pH). Simultaneously, the same research group developed a more advanced dual-stimulus (temperature and pH)-responsive HG system for the transdermal delivery of LT, a flavonoid (sub-class of flavonones) found in different plant extracts and pepper fruits, with extracts of *Lavandula angustifolia* as a major source [37]. LT, which structurally differs from Q only for the absence of the -OH group at C3 and biologically shares with Q the same wide range of activities, has recently received special attention due to its pronounced ability to inhibit keratinocytes hyperproliferation and to decrease NF-κB proinflammatory pathway in psoriatic skin [76]. To obtain this smart HG system, they synthesized a primary cross-linked HG of poly(*N*-isopropylacrylamide) (the temperature-sensitive unit) via free radical polymerization with *N*,*N*-methylene bisacrylamide as linker and sodium persulfate as initiator. To this first HG was then added a second polymeric network consisting in HA (the pH-sensitive unit), which was cross-linked in turn with divinyl sulfone via Michael addition. The resulting double cross-linked interpenetrating polymer network HGs were first characterized for their rheological, physicochemical and toxicity profiles, and were eventually loaded with a solution of the bioactive compound for further studies including drug release and skin permeability. In view of the obtained results (i.e., slow release at 37 °C and pH 7.4, which are similar to psoriatic skin conditions), this smart dual-responsive HG system can be envisaged as promising transdermal delivery carrier of LT for psoriasis skin relief [37].

The anti-inflammatory and anti-allergic properties of this flavonoid was evidenced by Szekalska M. et al. in multiple alginate-based micro-HGs loaded with CYN (Figure 3), that is, the naturally occurring 7-*O*-glucoside derivative of LT [62]. The glycoside was isolated from the extract of the aerial parts of *Bidens tripartita* and loaded into sodium alginate (which was chosen for its bioadhesive properties) HGs mixed with a variable amount of glycerol and/or propylene glycol. Several in vivo and ex vivo experiments proved that these CYN-loaded bioadhesive formulations reduce inflammatory cells in mouse skin with inflammation or atopic dermatitis and have a potential use for the treatment of psoriasis.

**Figure 4:**
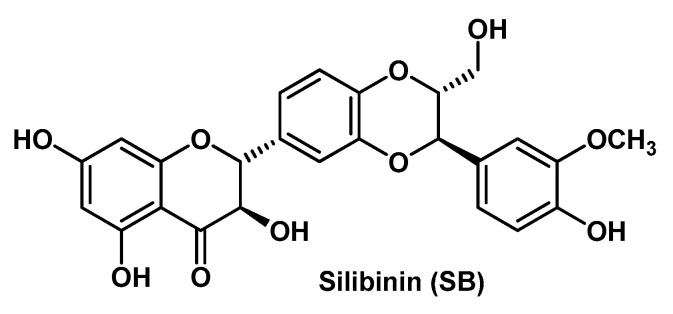
Chemical structure of Silibinin (SB).

Another flavonoid that constitutes the focus of current pharmaceutical technology efforts in view of its enormous therapeutic potential is SB (Figure 4). This flavonoid, structurally more complex than the other herein above reported parent compounds, is one of the most abundant and active components isolated from of the standardized extract of *Sylibum marianum* (milk thistle). Cruz’s research group developed a composite HG system, formed by SB-loaded pomegranate oil-based nanocapsules and a cross-linked co-polymer of acrylic acid and long chain methacrylates (i.e., Pemulen^®^ TR2) as a thickening agent, for the treatment of ICD [35]. In the design of this original HG system, the authors took also into account the additional biological activity of the oily component of the nanocapsules, as both SB and pomegranate seed oil exert anti-inflammatory activity, mostly by the inhibition of the NF-κB pathway [77,78]. The SB-loaded nanocapsules (formed by an oily core surrounded by a polymeric membrane) were prepared as a suspension according to a method carried out by the same research group, that is, the interfacial deposition of preformed polymer method [79]. Then, the final HG system was obtained by slow dispersion of this suspension into the co-polymer by using triethanolamine and imidazolidinyl urea (0.6%) for pH adjustment and preservative purposes, respectively. Physicochemical characterizations and in vivo anti-inflammatory assays fully confirmed the validity of this original composite HG system for cutaneous applications in the treatment of skin inflammatory disorders. The composite HG formulation showed superior skin retention and bioadhesive potential than the HG containing non-encapsulated SB. The same research group previously developed a similar HG system in which they incorporated the SB-loaded pomegranate oil-based nanocapsules in a gellan gum matrix [63]. Also in that case, the composite HG system turned out to be more effective in comparison to the SB-plain HG, specifically in protecting skin damage induced by UV irradiation.

Comparable outcomes (and then considerations) have been discussed by Shrotriya et al. in a research work where they used SB-loaded solid lipid nanoparticles incorporated into a Carbopol HG to afford the composite HG formulation that provided a controlled release of the bioactive compound and a superior therapeutic efficacy in a model of skin inflammation with respect to the HG formulation containing free SB [36]. Overall, these works performed on SB demonstrate the vast potential of the nanocomposite HGs as delivery systems for topical applications. SB is also quite active towards a variety of cancer types, including metastatic malignancies, as it is also able to inhibit pathways that play a key role in cancer progression and invasion. Among these oncogenic pathways inhibited by SB, one of the most important is the signal transducer and activator 3 (STAT3), which is also highly related to malignant melanoma [80]. In this regard, Makhmalzadeh B.S. and co-workers developed SB-loaded thermo-responsive HGs based on triblock co-polymers of poly[(α-benzyl carboxylate-ε-caprolactone)-co-(α-benzyl carboxylate-ε-caprolactone)]_ran_-*b*-PEG-*b*-poly[(α-benzyl carboxylate-ε-caprolactone)-co-(α-benzyl carboxylate-ε-caprolactone)]_ran_ (PCBCL-*b*-PEG-*b*-PCBCL), namely PolyGel^TM^ [38]. They obtained these co-polymers in a two-step process by functionalizing (ester junction) first the dihydroxy PEG at the two -OH termini with α-benzyl carboxylate-ε-caprolactone (BCL) to afford PBCL-*b*-PEG-*b*-PBCL, which was then randomly de-protected from the benzyl groups by catalytic (Pd/C 10%) hydrogenation to give the final co-polymer PolyGel^TM^. The HG preparation and SB loading were performed by adding in sequence PolyGel^TM^ and SB in a Tris-buffer (pH = 7.4) under magnetic stirring. For a comparative study, they prepared with the same method a SB-loaded HG based on Pluronic^®^ F-127, a commercially available triblock co-polymer of PEG and poly(propylene glycol). In principle, a block co-polymer comprises a linear arrangement of blocks (defined as portions of a polymeric molecule) with constitutional distinguishing features that may be exploited for a plethora of biomedical applications in nanotechnology. In vivo and ex vivo assessments showed that the newly synthesized SB-loaded thermo-responsive (sol-gel transition at 34–35 °C) HG inhibited the growth of B16-F10 melanoma cells and effectively suppressed pSTAT3 activity in these cells, thus suggesting that this formulation can provide valuable topical delivery of SB.

### 3.3. Hydrogels Containing Phenols with Trans-Cinnamic Acid Skeleton

**Figure 5:**
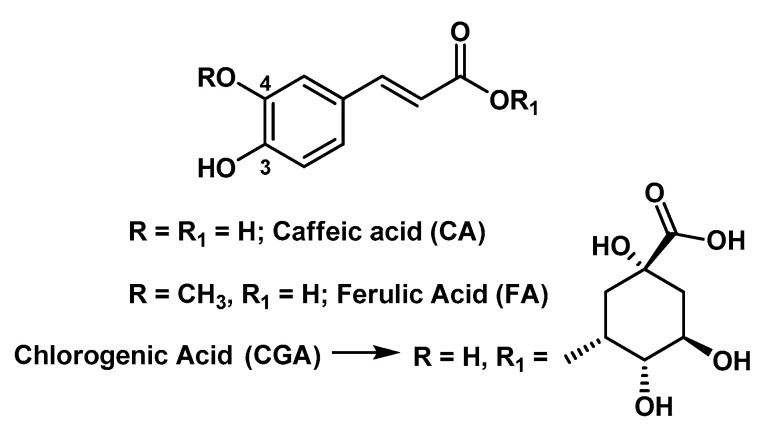
Chemical structure of natural phenols with *trans*-cinnamic acid skeleton.

Phenolic compounds endowed with a *trans*-cinnamic acid skeleton are present in high concentrations in leaves, fruits and vegetables. In particular, those with a *p*-hydroxy-*trans*-cinnamic motif have a huge potential as free radical scavengers in view of the fact that they may form phenoxy radicals which are highly stabilized by resonance. In this context, FA (4-hydroxy-3-methoxycinnamic acid; Figure 5) arises as one of most bioactive compounds. In addition to its primary and extraordinary antioxidant activity, FA also displays an extensive range of therapeutic properties, including anticancer, anti-inflammatory, antimicrobial, neuroprotective, radioprotective and antidiabetic [81]. As for flavonoids, this class of phenolic derivatives shows poor bioavailability due to its low water solubility and marked first-pass metabolism. Therefore, there is a high demand for the development of FA-containing formulations for an efficacious delivery of this bioactive compound.

Bairagi U. et al. proposed a Carbopol^®^ 980 HG system containing FA-loaded PLGA [poly(lactic-co-glycolic acid)] nanoparticles as potential tool in diabetic wound healing [28]. FA was loaded into the co-polymer PLGA with the nanoprecipitation method by using the surfactant Poloxamer 188 as a stabilizer. The nanoprecipitation technique represents a clean alternative for the preparation of delivery formulations. This technique consists of a slow addition under stirring of a non-solvent to a polymer solution (in this case acetone) containing the bioactive molecule. This procedure results in a decrease in the interfacial tension between the two phases and the rapid diffusion of the polymer solution into the non-solvent phase. The consequent increase in the surface area induces the formation of polymeric nanoparticles, which precipitate in a four-step mechanism (supersaturation, nucleation, growth by condensation, and growth by coagulation). The resulting FA-PLGA nanoparticles were then added to the gelling base, which was eventually neutralized with triethanolamine to afford the gelation. The in vivo studies (diabetic rats) indicated that FA, released from this HG system after topical application, promoted the wound healing.

Bai J. et al. developed FA-containing transdermal HG patches to treat skin damage induced by UV radiation [16]. The authors used a partially neutralized polyacrylate (NP700) as gelling base, and different combinations of dihydroxyaluminum aminoacetate, tartaric acid, glycerine, FA and water to obtain the HG with the best release rate and skin penetration performance. Dihydroxyaluminum aminoacetate acted as a cross-linking agent for the polymeric backbone by releasing aluminum ions in acidic conditions, promoted by tartaric acid in the presence of water. The antioxidant potential of FA, in relation to the development of effective delivery systems, has also been taken into consideration for the treatment of corneal wound healing, as low ocular bioavailability and short residence time are usually limiting factors associated with (poly)phenolic antioxidants topically administrated.

Tsai C.-Y. et al. investigated the in vitro and in vivo effects of FA in rabbit corneal epithelial cells and in a rabbit corneal alkali burn model, by developing a FA-loaded thermo-sensitive CS-based HG [40]. The system was obtained by adding in sequence G and GP to CS, then FA to the resulting solution to afford the gelation. This FA-HG formulation showed promising results both in vitro (by protecting corneal cells from oxidative stress and by reducing inflammation and apoptosis) and in vivo (by promoting corneal wound healing after alkali injury).

CA (3,4-dihydroxycinnamic acid; Figure 5) is another natural phenolic derivative endowed with a *trans*-cinnamic acid structural motif. It is found mainly in grapes, pomegranate and olive, and possesses a range of biological properties and applicability drawbacks that overlap those of FA. Pinho E. et al. prepared CD-based HGs for the controlled topical delivery of CA by direct cross-linking of CDs [i.e., β-CD and hydroxypropyl-β-CD (HPβ-CD)] with HPMC [41]. The synthetic strategy entailed the use a bifunctional linker (i.e., 1,4-butanediol diglycidyl ether) with epoxide end-groups able to react with the -OH groups of CDs and HPMC in alkaline medium. CA was then loaded into the resulting co-polymeric HGs by the swelling method. This system turned out to be promising for the treatment of wound infections, as they displayed relevant antimicrobial activity against three different bacterial strains, good physicochemical properties, such as superabsorbency and viscoelasticity, and low toxicity toward healthy cells (3T3 murine fibroblasts). Although the HPβ-CD-based HG showed a higher swelling property than β-CD-based HG, the latter resulted in a superior loading capacity due to a more suitable complexation of the bioactive compound with the β-CD.

CGA is the ester of CA with the 3-hydroxyl of l-quinic acid (Figure 5), which was found as the major component of the aqueous extract of the residual biomass of green coffee after oil extraction from coffee beans (coffee beans residual press cake) and was found to have beneficial effects on wound repair in mice models when incorporated in a Carbopol^®^ 940 HG [26].

### 3.4. Thymol-Containing Hydrogels

**Figure 6:**
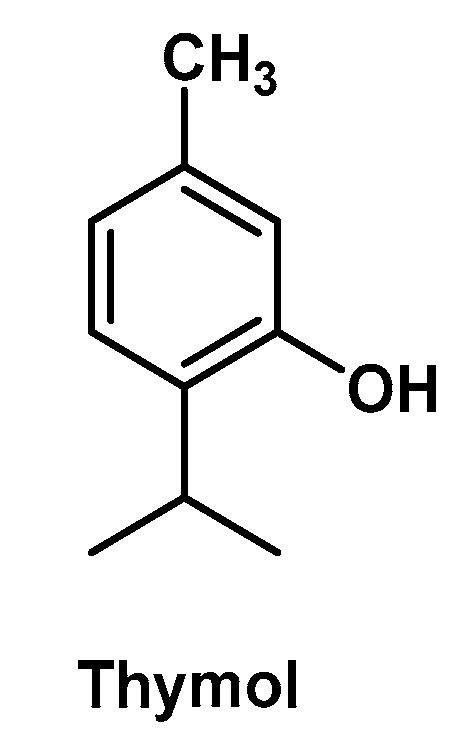
Chemical structure of thymol.

Thymol is a natural component of essential oil extracted from many plant varieties, such as *Thymus vulgaris*, *Lippia gracilis*, *Origanum vulgaris* and plants of the *Myrtaceae* family. It can be defined as a low molecular weight phenylpropanoid (also isoprenoid or terpenoid; Figure 6) and, as with many other structurally related constituents of essential oils (e.g., eugenol, carvacrol), is a secondary metabolite endowed with antimicrobial, antioxidant and anti-inflammatory activity. However, thymol has a low solubility in water and is a volatile compound—two factors that limit its development as a drug. Therefore, biocompatible delivery systems for this bioactive compound are in high demand. Alvarez Echazú M.I. et al. investigated the antimicrobial and antioxidant properties of the thymol by means of a CS-based HG designed for oral local delivery [43]. CS was selected as base polymer for several reasons: (i) it is a biodegradable and non-toxic scaffold which may undergo enzymatic hydrolysis within the oral cavity [82]; (ii) it is a mucoadhesive polymer due to its polycationic character that allows interaction with the mucin [83]; (iii) as previously stated, it possesses its own antimicrobial activity exerted by ionic interactions with the microorganism’s anionic cell surface proteins and/or a chelating mechanism [84]; (iv) it is a biomaterial suitable for oral tissue regeneration [85]. The CS-based HG was prepared by a spraying method by using a NaOH 1 N solution containing thymol to afford the sol-gel transition and a delivery system with the desired physicochemical and structural properties (e.g., swelling behavior, porosity, viscosity). The in vitro assessments proved the antimicrobial and antioxidant activity of this thymol-containing HG together with its biocompatibility. The authors proposed such a HG system as an alternative for periodontal disease treatment.

Thymol-containing HGs may be also successfully employed as wound dressing materials. In this regard, Jiji S. et al. developed a BCT-HG which turned out to be effective for wound healing in an in vivo burn model [34]. This thymol enriched HG system (which was obtained by soaking bacterial cellulose sheets (50% water) in a thymol water solution (1%)) also showed high biocompatibility towards fibroblasts and strong antimicrobial activity. In this case, the natural polymer does not possess any antimicrobial activity, which is then exclusively ascribed to an efficient release of thymol from the HG.

### 3.5. Plant Extract-Containing Hydrogels

Plants have been (and continue to be) a major source of medicine throughout human history. Therefore, plant extracts are still used worldwide as natural drugs in traditional folk medicine, especially for the treatment of several infectious diseases and as pain/inflammation relief. It is not uncommon to find that these natural complex drugs may have superior therapeutic benefits with respect to the single bioactive components. In this view, the development of delivery systems for these types of drugs constitutes an active area of the current pharmaceutical technology efforts.

In regard to the HG-based materials, Balestrin L.A. and co-workers proposed a delivery system composed of a nanoemulsion of *Achyrocline satureioides* crude ethanolic extract (80% *v*/*v*) and a Carbopol^®^ Ultrez 20 matrix as gelling agent [36]. The HG system was obtained by the spontaneous emulsification (oil-in-water) method and was intended as topical formulation for protection of the UV-induced skin damage. *Achyrocline satureioides* is a medical plant native to the Southeast region of South America which contains as its main bioactive compounds the antioxidant flavonoids Q, 3-Me-Q, and LT [86]. The advantage of this HG formulation with respect to the nanoemulsion is mainly due to its rheological properties (i.e., non-Newtonian pseudo-plastic behavior), which allow the incorporation of the extract in a suitable semi-solid pharmaceutical dosage form and its slow release. This study also evidenced that 3-Me-Q proved the highest skin permeation/retention performance which is related to both its higher initial concentration in the nanoemulsion and its higher partition coefficient than the other two flavonoids. Furthermore, the validity of this HG system (in comparison the nanoemulsion) was assessed by antioxidant activity studies aimed at quantifying the rate of carbonylated proteins and the residual free -SH of keratinocytes associated with UV-light radiation.

**Figure 7:**
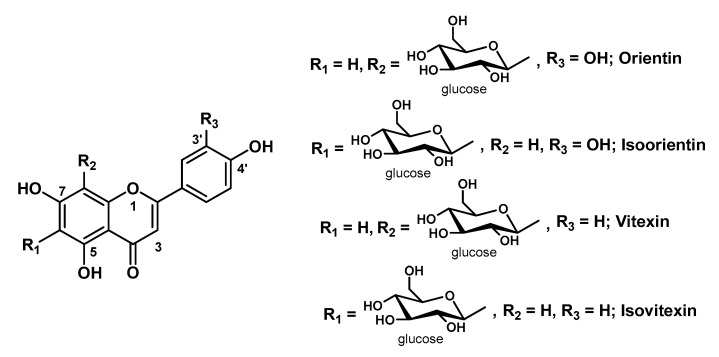
Chemical structure of some flavonoids of *Passiflora edulis* extract.

A CS-based HG containing a mixture of flavonoids extracted from *Passiflora edulis* Sims leaves was prepared by Soares’s research group (Figure 7) [31]. The HG was obtained by simple addition of glycerin to an acetic acid solution of the polymer, together with small amounts of methylparaben and propylparaben as preservatives. The HG matrix was then loaded with the bioactive component by adding PEG40 hydrogenate castor oil (Eumulgin^®^ CO 40) as a stabilizing agent and the dry plant extract (butanol fraction). This flavonoids-rich HG was designed as a wound dressing to be applied in the form of a thin film on the skin wound site and was successfully tested in an in vivo diabetic mice model. The flavonoids content of plant extract was estimated in Q, LT, APG, isoorientin, orientin, isovitexin and vitexin (Figure 7) (all endowed with marked antioxidant and anti-inflammatory properties). The in vitro studies confirmed the antioxidant activity of this formulation, which is also safe on fibroblasts and able to stimulate the antioxidant defense system in the initial phase of the healing process.

Another plant extract-containing HG with potential applicability for tissue restoration of skin wounds has been developed by Carmignan F. et al. [30]. The composition of the HG is simply made by Carbopol (1%), methylparaben (0.1%; preservative) and water as a vehicle, to which was added the ethanolic extract (2%) of *Equisetum pyramidale* shoots. This medicinal plant (also known as “cavalinha” or “horsetail”) is included in the Brazilian health care system as a valid and low-cost alternative for wound healing treatments. The phytochemical analysis performed by the authors on the ethanolic extract evidenced a wide variety of secondary metabolites, including polyphenolic derivatives (e.g., flavonoids), tannins, saponins, steroids, coumarins, triterpenes and reducing sugars. Therefore, the wound healing capacity of this plant extract and the related HG system is realistically ascribed to the bioactivity of more components which might also act synergistically. Presumably, the flavonoid (which are also the main constituents with pinocembrin as primary flavonoid) content plays a major role in the overall activity together with tannins, which are known for their ability in stimulating keratinocytes proliferation and favoring extracellular matrix formation.

**Figure 8:**
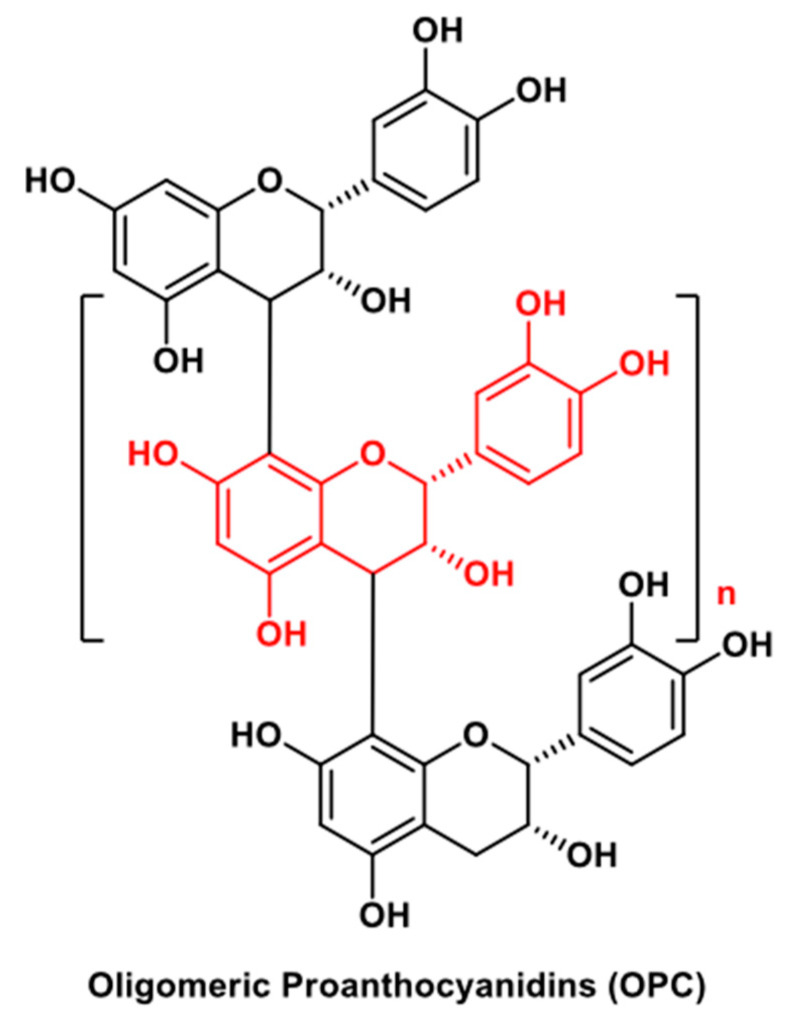
General structure of the oligomeric proanthocyanidins (OPC; *n* = 0–7).

An advanced light-responsive HG system containing OPC (Figure 8) for wound healing and melanoma therapy was developed by Ma H. et al. [29]. OPC are a mixture of oligomers of catechin units with a flavonoid-like core structure contained mainly in the grape-seed extracts, and widely used for their anticancer and antibacterial properties and also as cross-linking agents, including collagen-based HGs [87]. This smart system is formed by a HG scaffold whose precursors include a mixture of sodium alginate/Pluronic^®^ F-127, CaSiO_3_-nanowires, and l-(+)-glutamic acid. Sodium alginate and CaSiO_3_-nanowires constitute the base components of the system; l-(+)-glutamic acid was employed to provide a slightly acidic environment and promote the Ca^2+^ ion release that in turn promotes the gelation of the system by self-cross-linking of sodium alginate; the commercially available co-polymer Pluronic^®^ F-127 was added to make the HG base soft in order to be prepared by a 3D printing technique. The addition of OPC accelerated the gelation process (cross-linking agent) and favorably affected the overall characteristics of the system, i.e., morphology, mechanical performance, photothermal responsiveness, rheological properties. Notably, these HG scaffolds are light-responsive in the near-infrared wavelength range, which is compatible with minimal skin damage. In addition, this smart HG system showed excellent biocompatibility and bioactivity for melanoma therapy and wound healing in several in vitro and in vivo assessments.

The known anti-inflammatory activity and ulcer healing property of the medicinal plant licorice (*Glycyrrhiza glabra*) prompted Moghadamnia’s research group to develop bioadhesive patches for the treatment of recurrent aphthous stomatitis [44]. Licorice extracts are rich in bioactive flavonoids (primarily LQ), to which is ascribed the therapeutic potential of this herbal medicine. These patches were formulated from tragacanth gum and licorice chloroformic extract (1%), and then sealed in foil sachets to consent the application to the buccal mucosa in a non-tacky and non-adhesive dry form. Once applied, the patches swell in contact with the saliva, forming an adhesive HG which attaches to the mucosa. This in situ HG generation constitutes the main advantage of the management of the recurring ulcers associated with this pathological condition with respect the other topical formulations. Clinical studies assessed that this type of formulation was efficacious in reducing inflammation and promoting the aphthous ulcers’ healing process, which is partially due to the mechanical protection of the biopatches.

## 4. Injectable Hydrogels

Injectable HGs are formulations aimed at forming the HG system in the injury/diseased site after the injection of a polymer solution, which undergoes in situ gelation in response to internal/external stimuli that promote cross-linking processes. This type of formulation is enviable in those situations in which topical and systemic administration do not provide sufficient amount of drug to the target because of poor bioavailability or side effects.

Tran N.Q. et al. developed CS-based in situ-forming HGs containing RU as injectable dressings for dermal wound healing [25]. This intricate system was built in a multistep synthetic procedure by activating first the two end groups of PEG with *p*-nitrophenyl chloroformate (NPC). Then, NPC-PEG-NPC (joined by carbonate ester moieties) was partially substituted with tyramine (TY) to afford the tri-units framework NPC-PEG-TA through a carbamate linkage formation. The second activated end group of PEG was reacted with the free -NH2 groups of the CS to form the functionalized polymer CS-PEG-TY. In parallel, RU was transformed in RU-hemisuccinate (RU-Sa) at its end sugar moiety (ester linkage) and eventually the two components were joined by a coupling reaction (-*NHCO*-) between the residual free -NH2 groups of the CS and the pendant free -COOH groups of RU-Sa to obtain the desired soluble polymer RU-CS-PEG-TY. The HG formation was determined by an enzyme-catalyzed oxidation reaction that may occur in physiological conditions (pH = 7.4) and entailed the contemporary addition of HRP and H_2_O_2_. The oxidant system enzyme/H_2_O_2_ promotes the cross-linking between the TY phenolic moieties at the end of the functionalized polymer via C-C bond formation at the ortho positions or C-O bond formation between the carbon at the ortho positions and the phenolic oxygen. Both in vitro and in vivo studies demonstrated that these in situ-forming HGs that release RU significantly enhanced fibroblast proliferation and wound healing as compared to HGs without RU.

Diverse works on injectable thermo-sensitive HGs containing FA have been carried by Cheng Y.-H. et al. (Table 1). The most recent one dealt with CS-based HGs designed for the treatment of cardiovascular diseases [49]. Specifically, these smart HG systems were able to protect Cisd2^−/−^ CMs from oxidative stress-induced damage. Cisd2 is a mitochondrial outer membrane protein that plays a key role in controlling the lifespan of mammals. Its expression decreases during the normal aging process and its functionality may be hampered by ROS overproduction [88]. The FA-loaded HGs were obtained with the same synthetic procedure carried out by the authors in their herein reported works [40,89]. The change in the amount of the components, i.e., CS, G, GP and FA, provided the thermo-responsiveness to the HG system with a sol-gel transition that occur within 1 min at body temperature (37 °C). The obtained results show sustained FA release, increased cell viability, biocompatibility in in vivo models, and protection from oxidative stress induced in Cisd2^−/−^ CMs. A similar FA-loaded thermo-sensitive HG system was previously proposed by the same authors as an injectable formulation for the treatment of the early stage of intervertebral disc degeneration, which is mainly related to the degenerative process of the inner nucleus pulposus [89,90]. Later on, Cheng Y.-H. and co-workers improved the therapeutic performance of this last injectable HG by linking covalently (-*CONH*-) the bioactive component (FA) to G instead of loading it to the solution of CS/G/GP (as in the previous work) [47]. The covalent linkage between the free -NH2 groups of G and the -COOH group of FA did not compromise the sustained release of the bioactive compound and provided a thermo-sensitive HG system with lower gelation temperature and faster gelation process with respect to the previous one due to an increase in the hydrophobic interactions among the components. The same FA-loaded composition CS/G/GP/ employed by Cheng et al., which turned out to be effective towards Cisd2^−/−^ CM and nucleus pulposus cells, was adopted by Dong G.-C. and co-workers as an antioxidant and neuroprotective thermo-sensitive HG towards Neuro-2a cells [46]. The HG system in this case was intended to protect neuronal cells from further damage associated with secondary brain injury, a cascade of pathophysiological events triggered by the massive release of ROS and excitatory neurotransmitters, calcium influx, mitochondrial dysfunction, activation of pro-inflammatory cytokines, etc., which occur soon after the disruption of the brain tissue in primary traumatic injury. FA was physically cross-linked to the soluble polymeric matrix and efficiently released to Neuro-2a cells, which were protected from H_2_O_2_-induced oxidative stress and apoptosis. Furthermore, the gene expression analysis evidenced that this FA-containing HG system up-regulated the brain-derived neurotrophic factor gene expression and down-regulated the gene expression of several inflammatory and apoptosis markers.

A thermo-sensitive HG for the intra-articular delivery of Q was developed by Mok S.-W. et al. [48], as this flavonoid showed in vitro chondroprotective properties and anti-apoptotic effects on arthritic fibroblast-like synoviocytes [91,92]. This injectable system was composed of a polypeptide, i.e., l-poly(alanine) (PA), functionalized with methoxy-PEG (mPEG) and was synthesized by ring-opening polymerization of *N*-carboxy anhydride of l-alanine on α-methoxy-ε-amino PEG. The soluble polymer mPEG-PA was then loaded with the bioactive component and the resulting solution underwent gelation at physiological temperature. The Q-containing thermo-HG was experimentally employed in an osteoarthritis rat model, wherein they observed a delay of the progression of this degenerative joint disease in the knee and a relief of the disease-associated pain symptoms.

**Figure 9:**
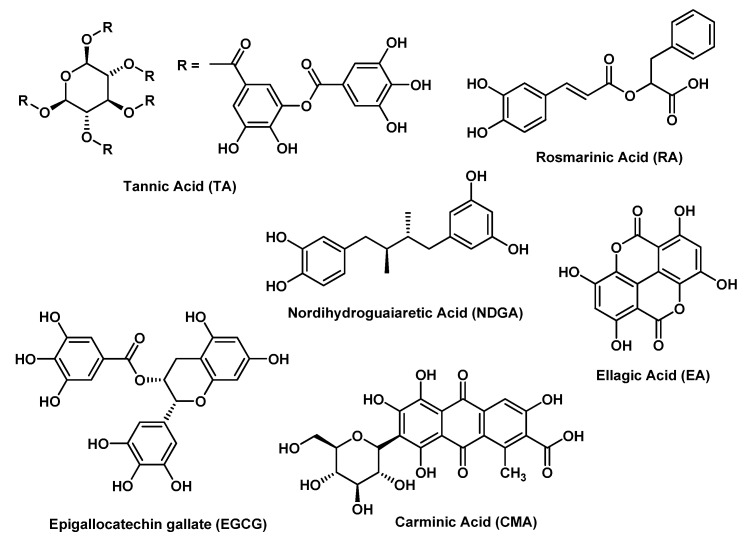
Chemical structure of natural polyphenols employed by Huang Z. et al. [43] for the preparation of injectable dynamic covalent HGs with boronic acid polymers.

Injectable HGs might also be responsive to pH values next to physiological conditions. This in situ sol-gel transition is quite challenging to achieve in a complex system. Huang Z. et al. proposed dynamic covalent HGs constituted of polymeric frameworks of PEG, functionalized with various phenylboronic acids and cross-linked by bioactive natural polyphenols such as tannic acid (TA), EGCG, rosmarinic acid (RA), RU trihydrate (RT), EA, nordihydroguaiaretic acid (NDGA) and carminic acid (CMA) (Figure 9) [45]. This strategy entailed the formation of a reversible boronic ester moiety, which is stable at alkaline pH and releases the free boronic acid moiety at acidic conditions. The equilibrium of these two forms can be tuned by selecting the appropriate boronic acid and bioactive polyphenol. The derivatized polymer was obtained through the oxidation of the terminal -OH groups of PEG into -COOH groups and coupling reaction of the latter with the aromatic free -NH2 of the selected phenylboronic acids. The covalent cross-links occur between the free boronic acid moiety of the functionalized polymer and the active vicinal diols (catechol groups) of the bioactive compounds. Stability studies showed that EA, EGCG and TA form stable HGs in physiological conditions. Moreover, EA-HG system displayed antiproliferative activity against CAL-27 human oral cancer cells, thus emerging as the best therapeutic candidate for further investigations.

## 5. Hydrogels Projected for Oral and Systemic Administration.

The oral administration route is particularly required for the treatment of chronic or long-term diseases. It may reduce costs and improve patient compliance and manageability as compared to injectable administration. HG-based formulations, in this case, must primarily protect the bioactive molecules from premature enzymatic degradation within the GI tract, and provide their controlled release to avoid local or systemic side effects. Stimuli-responsive HGs are of particular interest for oral delivery, as they can undergo modifications of their polymeric network in response to tunable environmental/external changes [93].

### 5.1. Quercetin-Containing Hydrogels

The vast majority of HG formulations for oral administration containing bioactive compounds from plant sources developed so far are Q-loaded systems. In this regard, Quagliariello’s group developed a HA-based HG as potential formulation for the treatment of medullary and papillary human thyroid cancer [52]. As said before, HA is a natural ligand of CD44 cell surface adhesion receptors [94], so the HA–CD44 interaction triggers an internalization process of HA by endocytosis after which HA is degraded within cytoplasm and the bioactive component released. The HG system was obtained with a solvent-non solvent method by using glutaraldehyde as a linker in acidic conditions. Then, the dried HG was loaded with Q by the soaking method. In addition, for comparative studies, the HG was also loaded with the Aurora kinases (types A and B) inhibitor SNS-314. Aurora proteins are serine/threonine kinases whose over-expression has been linked to the progression of different malignancies, including thyroid cancers [95]. The system was also loaded with a fluorescent probe (i.e., fluoresceinamine) for cellular up-take quantification. The results show that Q and SNS-314 may act synergistically against both types of thyroid cancer cells and that the release of Q from the HG occurs in a time- and CD44 interaction-dependent manner. Later on, the same research group proposed this active Q-loaded target-based HG towards hormone-responsive breast cancer cell line MCF-7 by adding as a potential synergic component the mTOR complex I allosteric inhibitor and rapamycin analogue RAD001 (Everolimus) [51]. In addition, in this case they found out that the two bioactive components act synergistically and accumulate into the cells in a time- and CD44 interaction-dependent manner. Moreover, this nano-HG system was effective in reducing the secretion of several inflammatory-related factors such as cytokines and metalloproteinases. Since great amounts of HA secretion and CD44 over-expression have been related to one of most malignant and aggressive form of brain tumors [96], that is, glioblastoma multiforme, Barbarisi et al. tested this Q-containing nano-HG system towards this type of cancer cells [50]. The HG was also loaded with the alkylating agent temozolomide, the first-line drug currently on the market for the treatment of glioblastoma multiforme. The same outcomes in terms of synergic antiproliferative effects between the two bioactive components, release modality, biocompatibility of the formulation, and ability of the HG to reduce cytokines production and other related tumor factors were detected.

To enhance the bioavailability of Q at an intended target site, Doosti M. et al. developed a magnetic-responsive nanocomposite HG based on starch grafted with fumaric acid [97]. The role of the fumaric acid in the system was then to improve the hydrophilicity of the base polymer, improve its loading capacity, and act as a cross-linking agent. To obtain the nanocomposite HG, starch was first grafted onto fumaric acid via radical polymerization by using ammonium persulfate as an initiator to achieve the co-polymer starch-fumaric acid. The latter was then added with a fixed mixture of iron salts (i.e., FeCl_3_·6H_2_O + FeCl_2_·4H_2_O) and NH_4_OH to afford the cross-linking process between the iron ions and the electron rich functional groups of the fumaric acid, the consequent HG formation and the simultaneous formation of the iron oxide nanoparticles within the system. The incorporation of Q into the prepared nanocomposite HG was performed by soaking procedures. The applicability of this system was assessed by in vitro and in vivo studies which proved effective Q release and increased Q bioavailability in the plasma and liver.

pH sensitivity assumes a remarkable part in the oral release of bioactive molecules, as substantial pH variability is found in different organs and body microenvironments. Therefore, HGs may react positively to these changes and enable the sustained and controlled release of their bioactive components accordingly. In this regard, Abdel Ghaffar A.M. and co-workers improved the performance of starch-based HGs by grafting the polymer onto acrylic acid via the direct gamma radiation technique [55]. This synthetic method offers some advantages, including the sterility of the product, the non-use of chemicals, the simultaneous cross-links and HG formation, and the ease of process control. The HGs were planned as pH-sensitive systems for the sustained and controlled release of RU at the colon after oral administration. Gel and swelling behavior of the poly(starch/acrylic acid)-based HGs increased by raising the amount of the grafting agent. The in vitro release studies proved that the system is highly pH-sensitive, with maximum release of the bioactive component in the range 6.8–7.7 (as in the colon). This is another enormous advantage of this formulation, as ordinary oral preparations prescribed for the treatment of chronic inflammatory disorders of the colon are largely absorbed at the upper part of the GI tract without reaching the active site. The in vivo evaluation also showed that this type of HG may represent a pivotal anti-inflammatory approach for the treatment of inflammatory bowel disease.

Another pH-sensitive and RU-loaded HG system was recently developed by Kunjiappan S. et al. [57]. The authors employed as a base polymer a class of alcohol soluble plant proteins isolated from maize endosperm, namely Zein. The protein was grafted with acrylic acid via the radical polymerization method by using *N*,*N*-methylene bisacrylamide as a cross-linking agent and ammonium persulfate as an initiator to afford a hybrid Zein-co-acrylic acid HG which was eventually loaded with RU plus the antitumor antimetabolite 5-FU by swelling equilibrium technique. The two bioactive components were efficiently loaded and then released (at pH 1.2 and 7.4) from the formulation, which also turned out to be significantly cytotoxic against two types of breast cancer cell lines. Taken together, these results suggest a potential application of this HG formulation as a potential vehicle for the oral delivery of anticancer drugs.

### 5.2. Hydrogels Containing other (Poly)Phenols

**Figure 10:**
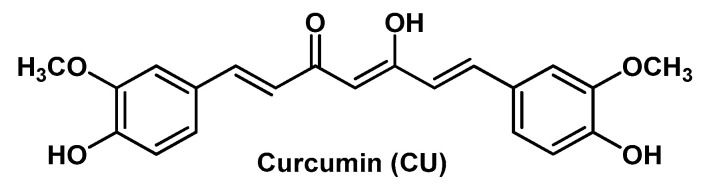
Chemical structure of curcumin (CU).

In addition to Q, other important natural (poly)phenols have been isolated from plants and employed for the preparation of HG formulations designed for oral administration. Among these, EGCG (Figure 9) and CU (Figure 10) take on great importance. EGCG is the most abundant and probably the most pharmacologically active flavonoid (sub-class of flavanols) contained in green tea leaves. CU is a diarylheptanoid (curcuminoid) found in plants of the ginger family (*Zingiberaceae*), mainly in the roots of *Curcuma longa* (turmeric). Both polyphenolic compounds have generated great interest for human health due to the wide range of biological properties that make them believed as panacea. Recent studied evidenced that both compounds may inhibit the aggregation of Aβ, then they could be beneficial in the prevention and treatment of Alzheimer’s disease [98,99]. The mechanism of the inhibition of the Aβ aggregation and remodeling of the Aβ fibrils is exerted by the two polyphenols according to two different pathways [100,101], a fact that suggests that they might act synergistically when in a suitable dual-delivery system. On this basis, Jiang Z. et al. developed a dual inhibitor-modified HA-nano HG wherein the two bioactive compounds were bound to the polymeric backbone via condensation chemistry (DCC + DMAP) [58]. EGCG-HA formed only dispersed HG structures, whereas CU-HA and CEHA self-assembled into nano-HGs. From this study, it clearly emerged that the two compounds act synergistically in inhibiting Aβ aggregation and increasing the cell viability of neuronal-like cell lines (i.e., SH-SY5Y).

The capacity of the (poly)phenols to interact and self-assemble with polymers was exploited by Hu B. and co-workers to develop reversible, shear thinning and thermo-resistant HGs, based on EGCG-binding amyloid fibrils and endowed with antibacterial activity [56]. The amyloid fibrils were prepared from lysozyme, globular peptidoglycans and lysostaphin M23 endopeptidase. They spontaneously self-assemble to form HGs by adding EGCG due to a mechanism that involves hydrophobic π-π stacking interactions and H-bond formation. Lysozyme, in its native monomeric format, possesses antibacterial activity and might provide additional antibacterial properties to the whole system. These hybrid formulations displayed strong antibacterial activity (with a mechanism initiated by membrane disintegration) against both Gram-(+) and Gram-(−) bacteria without any notable cytotoxicity towards human colonic epithelial cells, suggesting a potential application for the treatment of infection diseases of the small intestine.

The principle of the spontaneous self-assembly of polymeric structures was also adopted by Hu X. et al. for the preparation of a HG system for the oral delivery of green tea polyphenols [51]. In this case, the electrostatic interactions between two oppositely charged polysaccharides constituted the driving force directing the self-assembly process. The system was built from the anionic polysaccharide salecan and the cationic polysaccharide *N*,*N*,*N*-trimethyl CS through an eco-friendly solution mixing method. Salecan is a novel water-soluble linear β-1,3-glucan secreted by a salt-tolerant strain *Agrobacterium* sp. ZX09 that is receiving growing interest in the preparation of HGs [102]. The mixture of green tea polyphenols was eventually embedded into the HG system by the swelling method. This polyelectrolyte complex HG showed efficient release of the bioactive components in simulated GI fluids, with a rate that increases by increasing the pH (then, intestinal > gastric) and salecan/*N*,*N*,*N*-trimethyl CS ratio. These outcomes indicate a possible application of this formulation as a vehicle for the intestinal targeted delivery of this class of phytochemicals.

A promising pH-sensitive HG system for the oral delivery of bioactive compounds was developed by Zhao X. and Wang Z. It consists of a gellan gum-based HG containing a microemulsion (oil-in-water) of the flavonoid APG [53]. This composite HG showed controlled release of the bioactive component at acidic (pH 1.2; as in the stomach) and weak alkaline (pH 7.4; as in the small intestine) conditions. Under acidic medium, the strength of the cross-links turned out to be enhanced due to the decrease in the negative charges of the anionic polymer -COOH groups and the consequent reduction in the electrostatic repulsion between the polymer molecules. Under weak alkaline medium, the HG underwent slow degradation. Therefore, this pH-sensitive composite HG may be envisaged as an oral delivery system for the controlled release of hydrophobic molecules.

## 6. Conclusions

There has been in recent years a growing interest in the development of biocompatible and tunable delivery systems for bioactive molecules of natural source. In this regard, HGs are biomaterials with distinct properties that make them attractive for a wide range of biomedical and therapeutic applications. The base component of the HGs, i.e., the three-dimensional cross-linked polymeric network endowed with controllable swelling behavior in an aqueous medium, may be variously modified according to its chemical structure to match different requirements, such as active or passive targeting, loading capacity, controlled release of the bioactive components, mechanical strength, physicochemical properties, metabolic stability, etc., as well as to respond to different types of internal/external stimuli (i.e., pH, thermal, chemical, magnetic, light) in such a way so as to create smart delivery systems for drugs and biomolecules. On the other hand, plant-derived (poly)phenols, especially flavonoids and phenolic acids, are also gaining considerable attention due to their safety and huge therapeutic potential. However, the use of these phytochemicals as drugs is still hampered by their poor bioavailability due to low water solubility and marked first-pass metabolism. Taking this into account, HGs look like ideal, or at least sound among the most promising, delivery vehicles for this class of derivatives. This review highlights the extraordinary variability of the HGs projected to vehicle (poly)phenols, both regarding the chemical composition and the technologies employed. Many of these systems have been designed for a potential, effective employment in the treatment of skin and epithelial diseases, including not only skin wound healing but also contact dermatitis, burn injury, UV-erythema, psoriasis, etc., and, more interestingly, epithelial cancer. This is possible thanks to two factors. On the one hand, (poly)phenols are, as already mentioned, bioactive multifactorial agents, capable of acting as antioxidants and antimicrobials but also of modulating the main cellular signal pathways involved in inflammation and in cell replication and death; on the other hand, there is the possibility of producing HGs which are responsive to particular environmental conditions, such as pH. In addition to this, it should be emphasized that the innovative technologies used today could made it possible to use (poly)phenols in other biomedicine fields as well, through the possibility of being administered by injectable HGs (for example for the treatment of degenerative joint diseases) or by targeted HGs (for example, HGs based on the natural CD44 receptor ligand HA, potentially effective in the treatment of some types of cancer) or even by pH-responsive HGs that could allow (poly)phenols to overcome adverse conditions of the gastric environment (for example, in the case of colon-targeted delivery of these compounds).

However, one has to point out that almost all the results reported in this review were obtained in in vitro or ex vivo studies, or in vivo on experimental animals. Therefore, much caution should be used in extrapolating these findings to humans, and further studies should be directed to verify the efficacy as well as the safety and biocompatibility of the described systems.

## Figures and Tables

**Table 1:** Hydrogels for the delivery of plant-derived (poly)phenols.

Compound	System	Disease	Experimental Model		Reference
			In Vitro and Ex Vivo Models	In Vivo Models	
**Skin Wound Healing**					
**RU**	Carbopol Ultrez^®^ 10 NF HG	Skin wound healing		Skin wounds in Wistar rats	[24]
**RU**	CS-PEG-TY in presence of HRP and H_2_O_2_	Injectable wound dressing for skin wounds	Biocompatibility on L929 mouse fibroblasts	Skin wounds in Sprague Dawley rats	[25]
**CGA**	Carbopol 940 HG	Skin wound healing	Biocompatibility on L929 mouse fibroblasts	Skin wounds in Swiss mice	[26]
**Q**	Carbopol HG	Skin wound healing		Skin wounds in Sprague Dawley rats	[27]
**FA**	Carbopol 980 HG	Diabetic wound healing		Skin wounds in streptozotocin-diabetic Wistar rats	[28]
**OPC**	Light-responsive collagen-based HG	Wound healing	Regenerative capability on HUVECs and human dermal fibroblasts (HDFs)	Skin wounds in streptozocin-diabetic C57BL/6 mice	[29]
***Equisetum pyramidales* hoots extracts**	Carbopol HG	Wound healing		Skin wounds in Wistar rats	[30]
***Passiflora edulis* Sims leaves extracts**	CS-HG	Wound healing	Biocompatibility on L929 mouse fibroblasts	Skin wounds in alloxan- diabetic Wistar rats	[31]
**Q, GFs and 1-bromoperfluorooctane**	Carbopol 981 HG	Diabetic wound healing	Biocompatibility on HaCaT human keratinocytes and CCD-986sk human fibroblasts	Skin wounds in streptozotocin-diabetic C57BL/6 mice	[32]
**Q and OA**	HA-based nano-HG	Diabetic wound healing		Patients with diabetic foot ulcers	[33]
**Thymol**	BCT-HG	Wound healing	Antimicrobial activity against *Escherichia coli*, *Staphylococcus aureus*, *Pseudomonas aeruginosa*, *Klebsiella pneumoniae;* Biocompatibility on NIH 3T3 mouse fibroblasts	Wistar rats bearing third degree burn wounds	[34]
**Skin Permeation**					
**Q and RU**	Ceramide liposomes incorporated into cellulose HG using ECH as cross-linking agent	Improvement of drug skin permeation	Skin permeation study on ICR hairy albino mouse skin		[13]
**NR**	pH-responsive cl-CMC-g-pHEA	Improvement of drug skin permeation	Biocompatibility on HaCaT cells; Skin permeation study on hairless mouse skin		[14]
**LQG and LQ**	Ceramide Liposomes incorporated into cellulose HG	Improvement of drug skin permeation	Skin permeation study on dorsal skin of ICR hairy albino mice		[15]
**FA**	NP700 HG	Improvement of drug skin permeation	Skin permeation test on hairless mouse skin		[16]
**SB and pomegranate oil**	Pemulen^®^ TR2 HG system containing pomegranate oil-based nanocapsules	Improvement of drug skin permeation	Skin permeation study on human skin		[35]
**SB**	Carbopol 940 HG	Improvement of drug skin permeation	Skin diffusion study on human cadaver skin		[36]
**LT**	pH and temperature sensitive poly (*N*-isopropylacrylamide)/HA-based HG	Improvement of drug skin permeation	Skin permeation study on micropig dorsal skin		[37]
**SB**	Thermo-responsive hydrogels based on triblock co-polymers (PolyGel^TM^)	Improvement of drug skin permeation	Skin permeation study on mouse skin		[38]
**Epithelial Pathologies**					
**SB and pomegranate oil**	Pemulen^®^ TR2 HG system containing pomegranate oil-based nanocapsules	Irritant contact dermatitis		Croton oil-induced ear edema in Swiss mice; Cutaneous safety in humans	[35]
**SB**	Carbopol 940 HG	Irritant contact dermatitis		Skin irritancy in White New Zealand rabbits; Dinitrochlorobenzene - induced ICD in BALB/c mice	[36]
***Achyrocline satureioides* inflorescences extracts**	Carbopol^®^ Ultrez 20 HG	UVA/UVB radiation-induced skin damage	UVA/UVB light-induced oxidative stress on porcine ears skin		[39]
**FA**	Thermosensitive CS/G/GP-HG	Corneal wound healing	H_2_O_2_-induced oxidative stress in rabbit corneal epithelial CCL-60 cells	Corneal alkali burn in New Zealand albino rabbits	[40]
**LT**	pH and temperature sensitive poly (*N*-isopropylacrylamide)/HA-based HG	Psoriasis	Cytotoxicity assays on HaCaT human keratinocytes		[37]
**CA**	Cyclodextrin-based HG	Epithelial infections	Antibacterial activity against *Staphylococcus epidermidis*, *Staphylococcus aureus* and *Klebsiella pneumonia;* Proliferation study on NIH 3T3 mouse fibroblasts		[41]
**Q**	CHGZ-HGs	Skin and epithelial infections	Activity against *Staphylococcus aureus* and *Trichophyton rubrum;* Biocompatibility on L929 murine fibroblasts		[42]
**Thymol**	CS-HG	Periodontal diseases	Biocompatibility on NIH 3T3 mouse fibroblasts Antibacterial activity against *Streptococcus mutans* and *Staphylococcus aureus*		[43]
**Licorice (*Glycyrrhiza glabra*) extracts**	Tragacanth gum based HG	Recurrent aphthous stomatitis		Patients with aphthous ulcers	[44]
**Epithelial Cancer**					
**OPC**	Light-responsive collagen-based HG	Melanoma	B16F10 murine melanoma cells	Photothermal therapy in Balb/c mice bearing B16F10 cell tumor	[29]
**Q**	CHGZ-HGs	Skin cancer	Human skin carcinoma A431 cells		[42]
**SB (also in combination with doxorubicin)**	Thermo-responsive HG based on triblock co-polymers (PolyGel^TM^)	Melanoma	B16-F10 murine melanoma cells		[38]
**EA, EGCG, TA, NDGA, RT, RA, and CMA**	PEG-HG	Oral cancer	Human oral cancer CAL-27 cells		[45]
**Injectable and Targeted Hydrogels**					
**FA**	Thermosensitive CS/G/GP-HG	Secondary brain injury	H_2_O_2_-induced oxidative stress in Neuro-2a cells		[46]
**FA**	Thermosensitive CS/G/GP-HG	Intervertebral disc degeneration	H_2_O_2_-induced oxidative stress in nucleus pulposus cells from New Zealand rabbits		[47]
**Q**	Thermosensitive hydrogel based on mPEG-PA-HG	Osteoarthritis	Human chondrocytes from patients undergone knee arthroplasty	Rats undergone anterior cruciate ligament transection	[48]
**FA**	Thermosensitive CS/G/HG	Cardiovascular diseases	Cisd2-deficient (Cisd2^−/−^) cardiomyocytes and cardiac tissue of Cisd2 knockout mice	Biocompatibility in subcutaneously injected New Zealand albino rabbits and intramyocardially injected Cisd2 deficient (Cisd2^−/−^) and wild-type (Cisd2^+/+^)] rats	[49]
**Q (also in combination with temozolide)**	HA-HG	Glioblastoma multiforme	Human glioblastoma A172 and T98MG cells		[50]
**Q (also in combination with everolimus)**	HA-HG	Hormone-responsive human breast cancer	Human breast cancer MCF7 cells		[51]
**Q (also in combination with SNS-314)**	HA-HG	Medullary and papillary human thyroid cancer	Human medullary and papillary cancer thyroid B-CPAP and TT cells; Biocompatibility on NIH 3T3 mouse fibroblasts		[52]
**Hydrogels for Oral and Systemic Administration**					
**APG**	pH-sensitive gellan gum HGs	Oral delivery system for the controlled release of hydrophobic molecules	Drug release in different pH environments		[53]
**GTP**	Poly electrolyte complex HG	Oral delivery	Media simulating the gastric and the intestinal tracts		[54]
**RU**	pH-sensitive poly(starch/acrylic acid) HG	Ulcerative colitis		Dextran sulphate sodium-induced colitis in Wistar rats	[55]
**EGCG**	Polyphenol-binding amyloid fibrils self-assemble into reversible HGs	Infection of small intestine	Antibacterial activity against *Listeria monocytogenes*, methicillin resistant *Staphylococcus aureus*, *Streptococcus oralis*, *Escherichia coli, Klebsiella pneumoniae* and *Pseudomonas aeruginosa;* Biocompatibility on human colonic epithelial cells		[56]
**RU and 5-FU**	pH-responsive Zein-*co*-acrylic acid HGs	Anticancer activity	Breast cancer MDA-MB-231and MCF-7 cells		[57]
**EGCG and CU**	HA-HG	Alzheimer’s disease	Human neuroblastoma SH-SY5Y cells		[58]
